# Synthesis and crystal structure of (*Z*)-2-(6-chloroimidazo[1,2-*a*]pyridin-2-yl)-3-[4-(di­methyl­amino)phen­yl]acrylo­nitrile

**DOI:** 10.1107/S2056989025007248

**Published:** 2025-08-19

**Authors:** Ludovic Akonan, Eric Ziki, Deto Ursul Jean-Paul N’guessan, Songuigama Coulibaly, Mahama Ouattara, Viviane Adohi-Krou

**Affiliations:** a Laboratory of Fundamental and Applied Physics Nangui ABROGOUA University, Abidjan,Ivory Coast; b Laboratory of Matter Environmental and Solar Energy Sciences Research Team: Crystallography and Molecular Physics Félix Houphouët-Boigny University, Abidjan,Ivory Coast; c Department of Therapeutic Chemistry and Organic Chemistry UFR Pharmaceutical and Biological Sciences Félix Houphouët Boigny University, Abidjan,Ivory Coast; University of Hyogo, Japan

**Keywords:** crystal structure, π–π stacking, imidazo­pyridine, hydrogen bond

## Abstract

The structure of the title compound, C_18_H_15_ClN_4_, was determined at low temperature (100 K). In the crystal, the mol­ecules are connected through C—H⋯N and C—H⋯Cl inter­molecular hydrogen bonds generating a network that extend along the [010] direction. In addition, C—H⋯π and π–π stacking inter­actions as well as inter­molecular contacts contribute to the cohesion of the structure. Hirshfeld surface analysis indicates that the contributions to the surface for the H⋯H, H⋯N/ N⋯H, H⋯C/C⋯H, H⋯Cl/Cl⋯H and C⋯C contacts are 30.2, 19.9, 28.6, 12.2 and 3.7%, respectively.

## Abstract 2

The structure of the title compound was determined at 100 K. In the crystal, the mol­ecules are connected through C—H⋯N and C—H⋯Cl inter­molecular hydrogen bonds generating a network that extend along the [010] direction. I

## 1. Chemical context

Parasitic infections caused by gastrointestinal nematodes such as *Haemonchus contortus* represent a major challenge to the health of small ruminants, resulting in significant economic losses due to severe clinical symptoms, including diarrhoea, weight loss and increased mortality (Charlier *et al.*, 2014 ▸; Peter *et al., 2005 ▸;* Emery *et al.*, 2016 ▸).

Among the pharmacochemical strategies for developing new mol­ecules, the concept of mol­ecular juxtaposition is currently one of the fastest growing. It consists of combining two or more biologically active entities to obtain new biomolecules with high medicinal potential (Meunier, 2011 ▸). The application of this concept has led to the development of numerous drug mol­ecules, such as trioxaquines (anti­malarials), vancomycins (anti­biotics) and others. This so-called ‘two-shot gun’ strategy was developed with a view to reducing the emergence of drug-resistant germs (Meunier, 2011 ▸; Shaveta *et al.*, 2016 ▸).

As this research method has proved its worth, we adopted it to design a hybrid chemical profile resulting from the association of the imidazo­pyridine heterocycle and the acrylo­nitrile functional group. Indeed, acrylo­nitriles have emerged as a promising class of anthelmintic mol­ecules. In particular, 2-phenyl-3-(1*H*-pyrrol-2-yl)-acrylo­nitriles have demonstrated remarkable activity against *H. contortus*, with a lethal concentration (LD99) of 30 µ*M* (Gordon *et al.*, 2014 ▸).

Inspired by this work, we propose here an innovative structural design of 2-(6-chloro­imidazo[1,2-a]pyridin-2-yl)-3-phenyl­acrylo­nitrile derivatives. This design is based on the integration of an imidazo­pyridine core and a phenyl group within the acrylo­nitrile scaffold, with the aim of improving anthelmintic activity, metabolic stability and selectivity. The choice of imidazo­pyridine is justified by the fact that it is an isostere of benzimidazole, which is the pharmacophore carrier for several drugs used in therapeutics (Adachi *et al.*, 1969 ▸; Badgujar *et al.*, 2010 ▸; Balzarini *et al.*, 2005 ▸, 2006 ▸; Inuzuka *et al.*, 1976 ▸; Stevens *et al.*, 2003 ▸; Vieites *et al.*, 2008 ▸). The imidazo­pyridine core, in particular, offers a versatile chemical platform for specific inter­actions with parasitic targets, while the phenyl group enables pharmacokinetic properties to be modulated and affinity for parasitic receptors to be optimized.
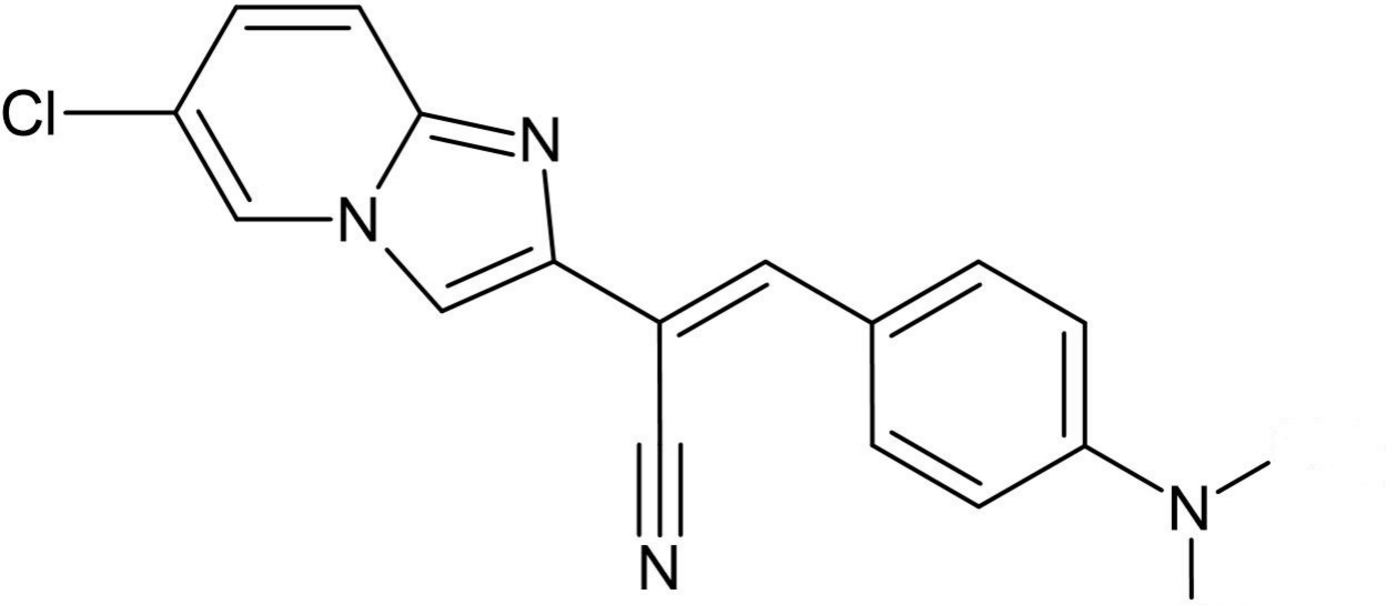


## 2. Structural commentary

**Figure 1:**
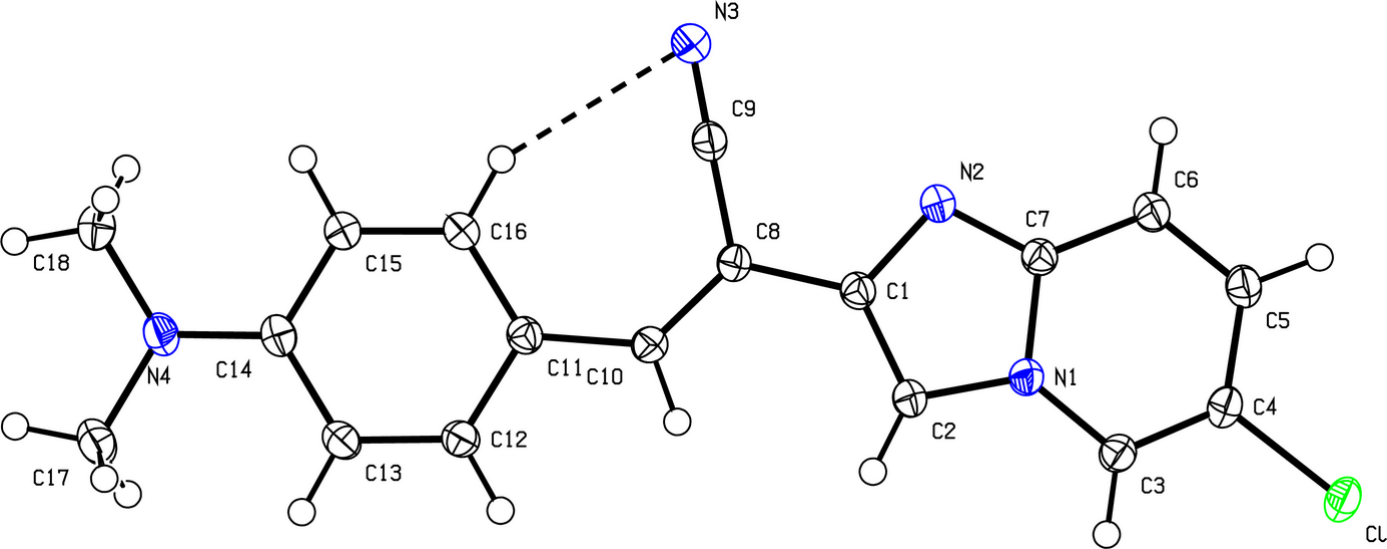
The mol­ecular structure of the title compound with displacement ellipsoids drawn at the 50% probability level. The dashed line indicates the hydrogen bond forming an *S*(7) pseudo-ring.

As shown in Fig. 1 ▸, the C1–C7/N1/N2/Cl 6-chloro­imidazo[1,2-*a*]pyridine moiety of the title mol­ecule is almost planar [r.m.s deviation = 0.036 (1) Å] and slightly inclined at an angle of 13.06 (5)° to the phenyl ring (C11–C16). A pseudo-ring with an *S*(7) motif is formed by atoms H16/C16/C11/C10/C9/C8/N3 as a result of the intra­molecular hydrogen bond (C16—H16⋯N3). An inspection of the bond lengths in the imidazo[1,2-*a*]pyridine ring shows that the N2—C7 [1.3302 (16) Å] and N2—C1 [1.3756 (16) Å] bond lengths are very different, suggesting that the electron density is preferentially located in the N2—C1 bond, as double-bond character, as seen in other imidazo­pyridine derivatives (Sissouma *et al.*, 2011 ▸). The length of N3—C9 [1.1502 (17) Å] indicates a double bond (Allen *et al.*, 1987 ▸). The C17 and N4 atoms of the di­methyl­amino group lie close to and on either side of the plane of the ring to which they are attached [deviations = 0.032 (1) and −0.036 (1) Å, respectively] whereas N4 is displaced by 0.243 (1) Å.

## 3. Supra­molecular features

**Figure 2:**
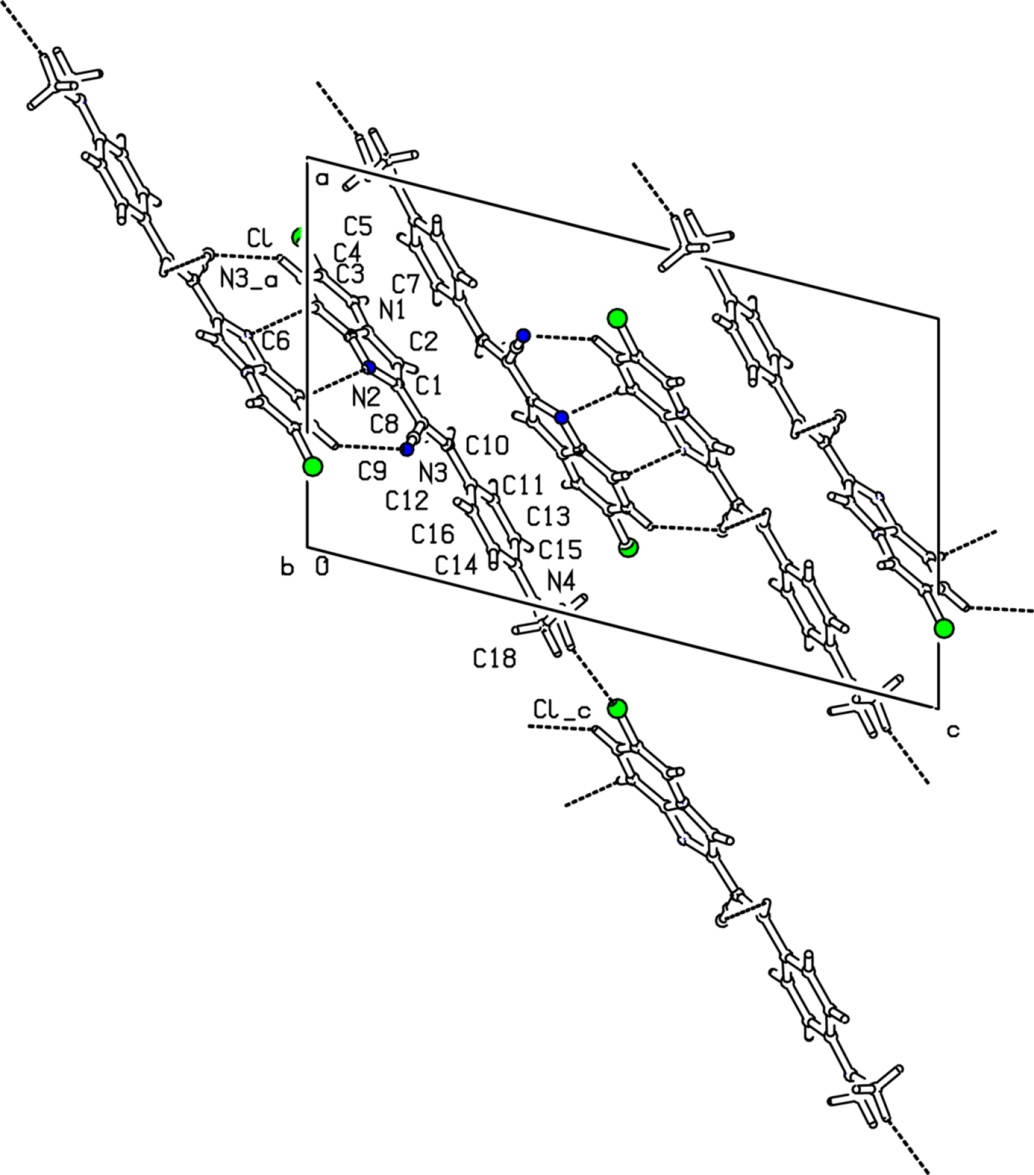
Partial packing diagram showing the [010] chains arising from C—H⋯N and C—H⋯Cl hydrogen bonds.

**Figure 3:**
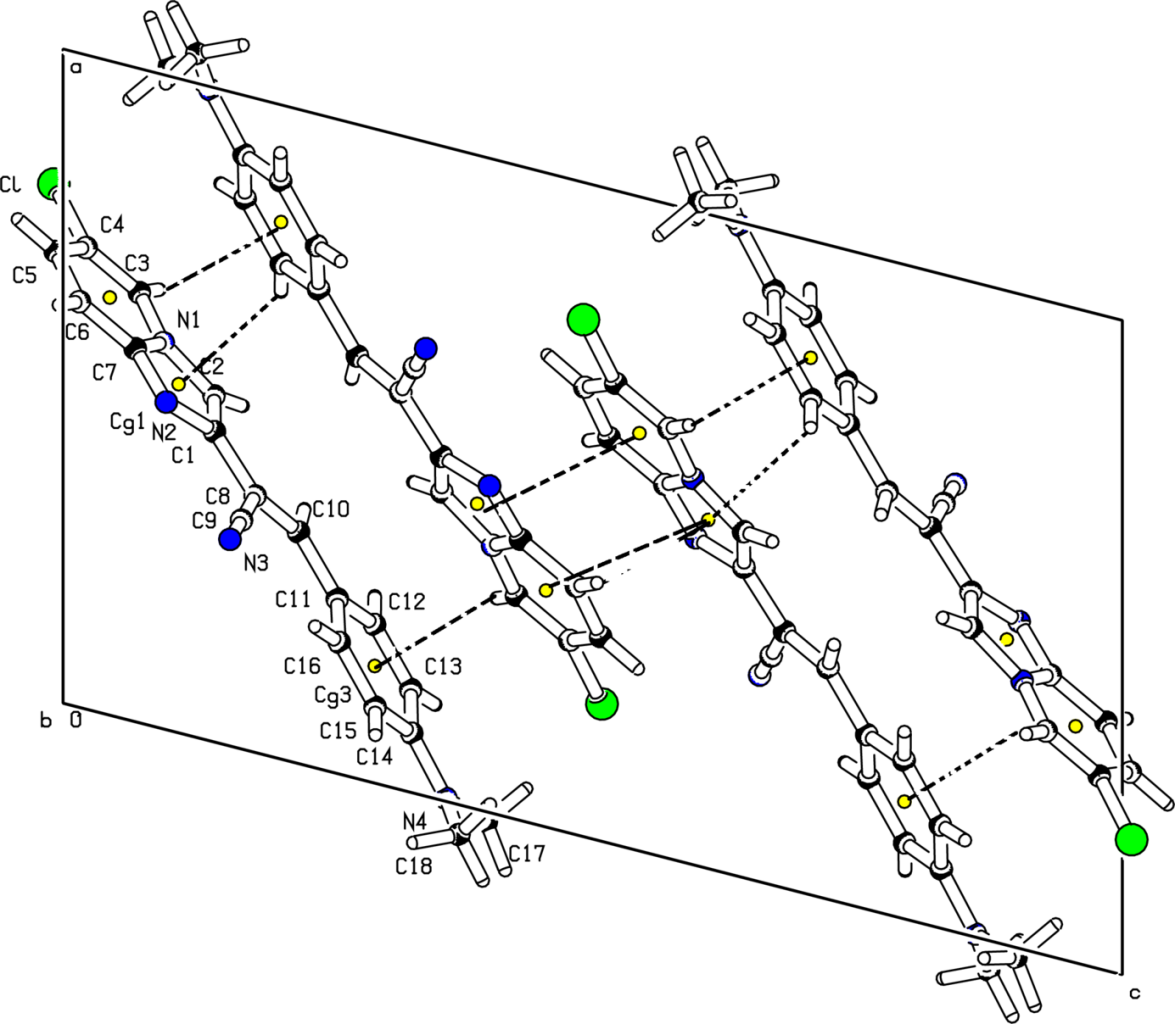
Partial packing diagram showing the π–π stacking and C—H⋯π inter­actions (dashed lines). The yellow dots are ring centroids.

In the crystal, cohesion is ensured by inter­molecular hydrogen bonds. These hydrogen bonds form a chain propagating along [010] axis direction with three adjacent 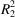
(9) and 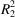
(8) loops between each pair of mol­ecules formed by the C5—H5⋯N3(−*x* + 1, −*y* + 2, −*z* + 1) and C6—H6⋯N2(−*x* + 1, −*y* + 2, −*z* + 1) hydrogen bonds (Fig. 2 ▸). Weak hydrogen bonds involving the chlorine atom Cl contribute to the consolidation of the crystal [C17—H17*B*⋯Cl(*x* + 1, −*y* + 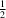, *z* − 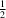), C17—H17*C*⋯Cl(−*x* + 1, *y* + 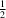, −*z* + 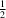)] (Fig. 2 ▸). Weak aromatic π–π stacking inter­actions are present between the pyridine (centroid *Cg*2) and imidazole (centroid *Cg*1) rings of symmetry-related (−*x* + 1, −*y* + 1, −*z* + 1) mol­ecules [centroid–centroid distance 3.5367 (8) Å], and also C—H⋯π inter­actions involving the phenyl (centroid *Cg*3) and imidazole rings (Table 1 ▸), forming a three-dimensional supra­molecular network (Fig. 3 ▸).

## 4. Hirshfeld surface analysis

**Figure 4:**
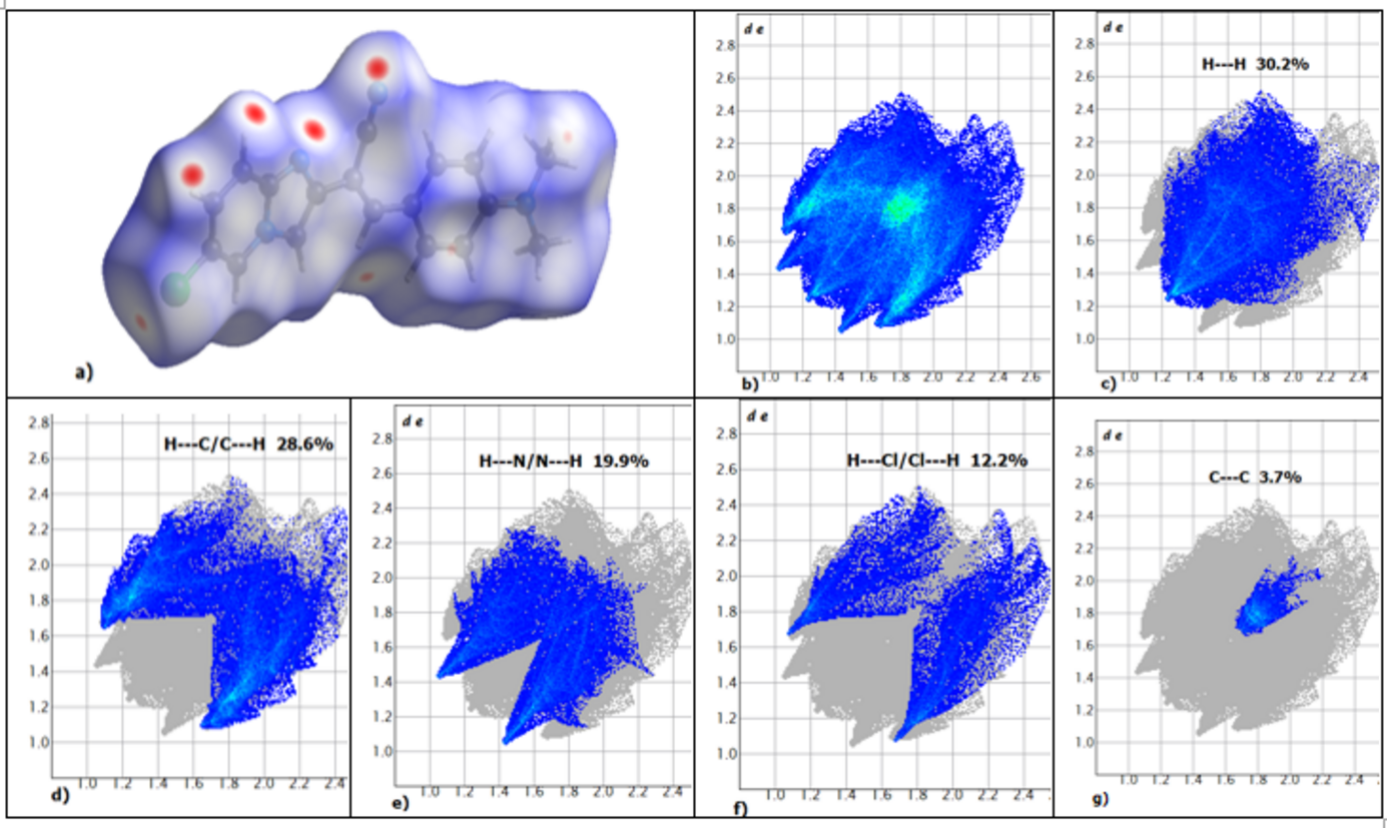
(*a*) Hirshfeld surface mapped over *d*_norm_ and two-dimensional fingerprint plots: (*b*) overall, and delineated into contributions from different contacts: (*c*) H⋯H, (*d*) H⋯C/C⋯H, (*e*) H⋯N/N⋯H, (*f*) H⋯Cl/Cl⋯H and (*g*) C⋯C.

The Hirshfeld surface and two-dimensional fingerprint (FP) plots (Rohl *et al.*, 2008 ▸) were generated by *CrystalExplorer17* (Spackman *et al.*, 2021 ▸). Intra­molecular and inter­molecular inter­actions were analysed by mapping the surface over *d*_norm_ where *d*_i_ and *d*_e_ are the contact distances from Hirshfeld surface to the nearest atom inside and outside, respectively. The contributions from different contacts are shown by partial analysis of the FP plots (Fig. 4 ▸). The π–π inter­molecular inter­actions correspond to C⋯C contacts. The largest contributions to the surface are made by H⋯H (30.2%, Fig. 4 ▸*b*) and H⋯C/C⋯H (28.6%, seen as red spots in Fig. 4 ▸*a*, FP plot in Fig. 4 ▸*c*) contacts. H⋯N/N⋯H and H⋯Cl/Cl⋯H contacts make contributions of 19.9% and 12.2%, respectively (Fig. 4 ▸*e*,*f*).

## 5. Database survey

A search of the Cambridge Structural Database (CSD version 5.45; Groom *et al.*, 2016 ▸) for compounds containing the chloro­imidazol and acrylo­nitrile moieties gave five hits [CSD refcodes APIFEL (Volovnenko *et al.*, 2009 ▸), BITSAA (Hranjec *et al.*, 2012 ▸), AZURAP (Zhao & Ng, 2011 ▸), HUBTOQ (Kusy *et al.*, 2019 ▸) and ABOFEG (Zhou *et al.*, 2021 ▸)].

## 6. Synthesis and crystallization

To a solution of 2.35g (24.9 mmol, 1 eq.) of 2-amino 4-chloro­pyridine in 25 ml of aceto­nitrile were added 3.2 g (25.2 mmol, 1.01 eq.) of 1,3-di­chloro acetone. The mixture was left to stir at room temperature for 12 h. The precipitate formed was isolated by vacuum filtration, washed with 2 × 15 ml of aceto­nitrile, filtered and dried at room temperature. The residue was then dissolved in 60 ml of water and the solution neutralized with a saturated solution of sodium hydrogen carbonate (NaHCO_3_). Impurities were extracted from the mixture with 2 × 15 ml of ethyl acetate; the aqueous phase was then kept refrigerated (278 K) and the product precipitated after 1 h. After vacuum filtration, 2-chloro­methyl imidazo[1,2-*a*]pyridine was isolated as a flaky white solid in 49.28% yield.

A mixture of 2-chloro­methyl imidazo[1,2-*a*]pyiridine (1 g; 6 mmol; 1 eq.) and potassium cyanide (0.43 g; 6.6 mmol; 1.1 eq.) was stirred for 12 h at room temperature in a 100 ml flask containing 10 ml of DMSO. The brown liquid was extracted with di­chloro­methane (2 × 50 ml), then washed with 2 × 50 ml of water. The organic phase was dried over magnesium sulfate, filtered and concentrated *in vacuo*. The brown paste formed crystallized after 30 minutes at room temperature in 87.23% yield, as 2-(imidazo[1,2-*a*]pyridin-2-yl)aceto­nitrile (N’Guessan *et al.*, 2025 ▸).

To a solution of 0.5 g (3.18 mmol; 1 eq.) of 2-(imidazo[1,2-*a*]pyridin-2-yl) aceto­nitrile in 8 ml of anhydrous ethanol, were added 5 drops of piperidine and 3.2 g (3.5 mmol; 1.1 eq.) of 4-(di­methyl­amino)­benzaldehyde. The mixture was refluxed for 12 h. The precipitate formed was isolated by vacuum filtration, washed with 10 ml of cold methanol, squeezed dry and then dried at room temperature. (*Z*)-2-(6-Chloro­imidazo[1,2-*a*]pyridin-2-yl)-3-[4-(di­methyl­amino)­phen­yl]acrylo­nitrile was isolated as a lumpy brown powder in 76% yield.

Crystallization was performed under ambient conditions by slow solvent evaporation. Approximately 30 mg of the compound were dissolved in 1 mL of cooled methanol and transferred into a 10 mL glass vial. The vial was sealed with aluminium foil pierced with five small holes using a needle, allowing the solvent to evaporate gradually while limiting dust contamination. The setup was kept undisturbed at room temperature (298 K) on a laboratory bench. After 10 days, well-formed crystals suitable for analysis were obtained. The crystals were then collected and dried in an oven at 313 K for 3 days to remove any residual solvent; m.p. = 469–472 K. ^1^H NMR (300 MHz, CDCl_3_) δ: 8.19 (*s*, 2H), 7.91 (*s*, 1H), 7.88 (*s*, 1H), 7.85 (*s*, 1H), 7.56 (*d*, *J* = 9.6 Hz, 1H), 7.47–7.45 (*m*, 1H), 7.44–7.41 (*m*, 1 H), 7.26 (*dd*, *J* = 9.6, 1.9 Hz, 1H). ^13^C NMR (75 MHz, CDCl_3_) δ: 130.9, 129.5, 124.0, 117.6, 111.6.

## 7. Refinement details

Crystal data, data collection and structure refinement details are summarized in Table 2 ▸. H atoms were positioned geometrically (C—H = 0.95–0.98 Å) and refined as riding with *U*_iso_(H) = 1.2–1.5*U*_eq_(C).

## Supplementary Material

Crystal structure: contains datablock(s) I. DOI: 10.1107/S2056989025007248/ox2017sup1.cif

Structure factors: contains datablock(s) I. DOI: 10.1107/S2056989025007248/ox2017Isup2.hkl

Supporting information file. DOI: 10.1107/S2056989025007248/ox2017Isup3.cml

CCDC reference: 2480418

Additional supporting information:  crystallographic information; 3D view; checkCIF report

Additional supporting information:  crystallographic information; 3D view; checkCIF report

## Figures and Tables

**Table 1:** Hydrogen-bond geometry (Å, °) *Cg*1 and *Cg*3 are the centroids of the N1/C2/C1/N2/C7 and C11–C16 rings, respectively.

*D*—H⋯*A*	*D*—H	H⋯*A*	*D*⋯*A*	*D*—H⋯*A*
C5—H5⋯N3^i^	0.95	2.58	3.3871 (17)	143
C17—H17*C*⋯Cl^ii^	0.98	2.96	3.6211 (15)	126
C6—H6⋯N2^i^	0.95	2.59	3.4554 (16)	151
C16—H16⋯N3	0.95	2.57	3.4275 (17)	151
C3—H3⋯*Cg*3^iii^	0.95	2.67	3.3054 (13)	125
C12—H12⋯*Cg*1^iii^	0.95	2.80	3.5101 (14)	132

Symmetry codes: (i) 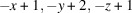; (ii) 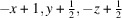; (iii) 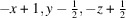.

**Table 2:** Experimental details

Crystal data	
Chemical formula	C_18_H_15_ClN_4_
*M*_r_	322.79
Crystal system, space group	Monoclinic, *P*2_1_/*c*
Temperature (K)	100
*a*, *b*, *c* (Å)	12.1985 (7), 6.2726 (3), 20.3813 (11)
β (°)	104.379 (2)
*V* (Å^3^)	1510.65 (14)
*Z*	4
Radiation type	Mo *K*α
μ (mm^−1^)	0.26
Crystal size (mm)	0.30 × 0.10 × 0.10

Data collection	
Diffractometer	Bruker D8 Venture
No. of measured, independent and observed [*I* > 2σ(*I*)] reflections	79364, 6055, 4535
*R*_int_	0.057
(sin θ/λ)_max_ (Å^−1^)	0.784

Refinement	
*R*[*F*^2^ > 2σ(*F*^2^)], *wR*(*F*^2^), *S*	0.048, 0.137, 1.09
No. of reflections	6055
No. of parameters	210
H-atom treatment	H-atom parameters constrained
Δρ_max_, Δρ_min_ (e Å^−3^)	0.41, −0.56

Computer programs: *APEX4* and *SAINT* (Bruker, 2019 ▸), *SHELXT2018/2* (Sheldrick, 2015*a* ▸), *SHELXL2018/3* (Sheldrick, 2015*b* ▸), *PLATON* (Spek, 2020 ▸) and *publCIF* (Westrip, 2010 ▸).

## supplementary crystallographic information

### (Z)-2-(6-Chloroimidazo[1,2-a]pyridin-2-yl)-3-[4-(dimethylamino)phenyl]prop-2-enenitrile Crystal data

**Table:**

C_18_H_15_ClN_4_	*F*(000) = 672
*M**_r_* = 322.79	*D*_x_ = 1.419 Mg m^−^^3^
Monoclinic, *P*2_1_/*c*	Melting point: 469 K
Hall symbol: -P 2ybc	Mo *K*α radiation, λ = 0.71073 Å
*a* = 12.1985 (7) Å	Cell parameters from 6055 reflections
*b* = 6.2726 (3) Å	θ = 2.1–33.9°
*c* = 20.3813 (11) Å	µ = 0.26 mm^−^^1^
β = 104.379 (2)°	*T* = 100 K
*V* = 1510.65 (14) Å^3^	Prism, yellow
*Z* = 4	0.30 × 0.10 × 0.10 mm

### (Z)-2-(6-Chloroimidazo[1,2-a]pyridin-2-yl)-3-[4-(dimethylamino)phenyl]prop-2-enenitrile Data collection

**Table:**

Bruker D8 Venture diffractometer	4535 reflections with *I* > 2σ(*I*)
Radiation source: Fine-focus sealed tube	*R*_int_ = 0.057
Mirror monochromator	θ_max_ = 33.9°, θ_min_ = 2.1°
π and ω scan	*h* = −19→18
79364 measured reflections	*k* = −9→9
6055 independent reflections	*l* = −30→31

### (Z)-2-(6-Chloroimidazo[1,2-a]pyridin-2-yl)-3-[4-(dimethylamino)phenyl]prop-2-enenitrile Refinement

**Table:**

Refinement on *F*^2^	Primary atom site location: structure-invariant direct methods
Least-squares matrix: full	Secondary atom site location: difference Fourier map
*R*[*F*^2^ > 2σ(*F*^2^)] = 0.048	Hydrogen site location: inferred from neighbouring sites
*wR*(*F*^2^) = 0.137	H-atom parameters constrained
*S* = 1.09	*w* = 1/[σ^2^(*F*_o_^2^) + (0.0678*P*)^2^ + 0.5322*P*] where *P* = (*F*_o_^2^ + 2*F*_c_^2^)/3
6055 reflections	(Δ/σ)_max_ < 0.001
210 parameters	Δρ_max_ = 0.41 e Å^−^^3^
0 restraints	Δρ_min_ = −0.56 e Å^−^^3^

### (Z)-2-(6-Chloroimidazo[1,2-a]pyridin-2-yl)-3-[4-(dimethylamino)phenyl]prop-2-enenitrile Special details

**Table:**

Geometry. All esds (except the esd in the dihedral angle between two l.s. planes) are estimated using the full covariance matrix. The cell esds are taken into account individually in the estimation of esds in distances, angles and torsion angles; correlations between esds in cell parameters are only used when they are defined by crystal symmetry. An approximate (isotropic) treatment of cell esds is used for estimating esds involving l.s. planes.

### (Z)-2-(6-Chloroimidazo[1,2-a]pyridin-2-yl)-3-[4-(dimethylamino)phenyl]prop-2-enenitrile Fractional atomic coordinates and isotropic or equivalent isotropic displacement parameters (Å^2^)

**Table:**

	*x*	*y*	*z*	*U*_iso_*/*U*_eq_	
Cl	0.21001 (3)	0.25461 (5)	0.50877 (2)	0.02489 (9)	
N1	0.40970 (9)	0.51243 (16)	0.40546 (5)	0.01837 (19)	
N2	0.49924 (9)	0.82472 (17)	0.40256 (5)	0.01965 (19)	
N3	0.68410 (11)	1.12761 (18)	0.34242 (6)	0.0268 (2)	
N4	0.99529 (10)	0.63550 (18)	0.13872 (6)	0.0239 (2)	
C2	0.47112 (10)	0.4903 (2)	0.35756 (6)	0.0197 (2)	
H2	0.475218	0.368416	0.330590	0.024*	
C12	0.75773 (11)	0.44782 (19)	0.20603 (6)	0.0197 (2)	
H12	0.714509	0.321705	0.205787	0.024*	
C3	0.34252 (11)	0.3676 (2)	0.42803 (6)	0.0205 (2)	
H3	0.330171	0.228287	0.409333	0.025*	
C9	0.65039 (11)	0.9566 (2)	0.33157 (6)	0.0206 (2)	
C11	0.73315 (10)	0.62767 (19)	0.24120 (6)	0.0185 (2)	
C8	0.60778 (10)	0.74328 (18)	0.31874 (6)	0.0177 (2)	
C4	0.29460 (10)	0.4323 (2)	0.47822 (6)	0.0204 (2)	
C14	0.90927 (10)	0.6321 (2)	0.17091 (6)	0.0201 (2)	
C13	0.84261 (11)	0.4480 (2)	0.17179 (6)	0.0212 (2)	
H13	0.856220	0.323192	0.148631	0.025*	
C10	0.64595 (10)	0.60587 (19)	0.27785 (6)	0.0189 (2)	
H10	0.608870	0.471481	0.272285	0.023*	
C7	0.42980 (10)	0.71865 (19)	0.43190 (6)	0.0185 (2)	
C1	0.52549 (10)	0.68329 (19)	0.35718 (6)	0.0183 (2)	
C5	0.31255 (11)	0.6378 (2)	0.50741 (6)	0.0221 (2)	
H5	0.278109	0.677146	0.542630	0.026*	
C15	0.88371 (10)	0.8148 (2)	0.20542 (6)	0.0205 (2)	
H15	0.925936	0.941883	0.205144	0.025*	
C16	0.79884 (10)	0.8120 (2)	0.23939 (6)	0.0200 (2)	
H16	0.784285	0.937137	0.262090	0.024*	
C17	1.01552 (12)	0.4510 (2)	0.10050 (7)	0.0276 (3)	
H17A	1.028965	0.325721	0.130162	0.041*	
H17B	1.081974	0.476797	0.082732	0.041*	
H17C	0.949284	0.425717	0.062784	0.041*	
C6	0.37982 (11)	0.7796 (2)	0.48461 (6)	0.0218 (2)	
H6	0.392841	0.917871	0.504016	0.026*	
C18	1.04551 (12)	0.8369 (2)	0.12625 (7)	0.0270 (3)	
H18A	0.987633	0.926490	0.096997	0.041*	
H18B	1.106674	0.809941	0.103943	0.041*	
H18C	1.076080	0.910152	0.169393	0.041*	

### (Z)-2-(6-Chloroimidazo[1,2-a]pyridin-2-yl)-3-[4-(dimethylamino)phenyl]prop-2-enenitrile Atomic displacement parameters (Å^2^)

**Table:**

	*U*^11^	*U*^22^	*U*^33^	*U*^12^	*U*^13^	*U*^23^
Cl	0.02422 (15)	0.02859 (16)	0.02381 (16)	−0.00599 (11)	0.00966 (11)	0.00203 (11)
N1	0.0195 (4)	0.0193 (4)	0.0171 (4)	−0.0016 (3)	0.0061 (3)	−0.0007 (3)
N2	0.0212 (5)	0.0199 (4)	0.0193 (5)	−0.0007 (4)	0.0078 (4)	−0.0013 (4)
N3	0.0333 (6)	0.0218 (5)	0.0295 (6)	−0.0022 (4)	0.0157 (5)	−0.0028 (4)
N4	0.0243 (5)	0.0243 (5)	0.0264 (5)	0.0033 (4)	0.0124 (4)	−0.0007 (4)
C2	0.0220 (5)	0.0206 (5)	0.0180 (5)	−0.0015 (4)	0.0081 (4)	−0.0016 (4)
C12	0.0225 (5)	0.0182 (5)	0.0188 (5)	0.0004 (4)	0.0057 (4)	−0.0006 (4)
C3	0.0218 (5)	0.0205 (5)	0.0197 (5)	−0.0025 (4)	0.0063 (4)	−0.0001 (4)
C9	0.0220 (5)	0.0215 (5)	0.0200 (5)	0.0009 (4)	0.0087 (4)	0.0002 (4)
C11	0.0199 (5)	0.0189 (5)	0.0172 (5)	0.0012 (4)	0.0054 (4)	0.0005 (4)
C8	0.0184 (5)	0.0188 (5)	0.0165 (5)	−0.0004 (4)	0.0056 (4)	0.0009 (4)
C4	0.0189 (5)	0.0243 (5)	0.0186 (5)	−0.0026 (4)	0.0057 (4)	0.0019 (4)
C14	0.0190 (5)	0.0234 (5)	0.0185 (5)	0.0031 (4)	0.0056 (4)	0.0004 (4)
C13	0.0245 (6)	0.0198 (5)	0.0203 (5)	0.0029 (4)	0.0073 (4)	−0.0019 (4)
C10	0.0206 (5)	0.0178 (5)	0.0188 (5)	−0.0008 (4)	0.0059 (4)	0.0003 (4)
C7	0.0188 (5)	0.0195 (5)	0.0174 (5)	−0.0009 (4)	0.0048 (4)	−0.0011 (4)
C1	0.0193 (5)	0.0193 (5)	0.0168 (5)	0.0001 (4)	0.0053 (4)	−0.0003 (4)
C5	0.0215 (5)	0.0265 (6)	0.0195 (5)	−0.0007 (4)	0.0076 (4)	−0.0017 (4)
C15	0.0212 (5)	0.0203 (5)	0.0211 (5)	−0.0007 (4)	0.0072 (4)	−0.0015 (4)
C16	0.0217 (5)	0.0194 (5)	0.0200 (5)	0.0002 (4)	0.0074 (4)	−0.0023 (4)
C17	0.0296 (6)	0.0289 (6)	0.0275 (6)	0.0052 (5)	0.0129 (5)	−0.0019 (5)
C6	0.0227 (5)	0.0234 (5)	0.0210 (5)	−0.0018 (4)	0.0086 (4)	−0.0036 (4)
C18	0.0244 (6)	0.0296 (6)	0.0300 (7)	−0.0017 (5)	0.0123 (5)	−0.0011 (5)

### (Z)-2-(6-Chloroimidazo[1,2-a]pyridin-2-yl)-3-[4-(dimethylamino)phenyl]prop-2-enenitrile Geometric parameters (Å, º)

**Table:**

Cl—C4	1.7360 (13)	C8—C10	1.3590 (17)
N1—C3	1.3769 (16)	C8—C1	1.4674 (17)
N1—C2	1.3774 (15)	C4—C5	1.4140 (18)
N1—C7	1.3994 (15)	C14—C13	1.4147 (18)
N2—C7	1.3302 (16)	C14—C15	1.4192 (17)
N2—C1	1.3756 (16)	C13—H13	0.9500
N3—C9	1.1503 (17)	C10—H10	0.9500
N4—C14	1.3690 (16)	C7—C6	1.4131 (17)
N4—C17	1.4500 (17)	C5—C6	1.3674 (18)
N4—C18	1.4539 (18)	C5—H5	0.9500
C2—C1	1.3815 (17)	C15—C16	1.3811 (17)
C2—H2	0.9500	C15—H15	0.9500
C12—C13	1.3848 (17)	C16—H16	0.9500
C12—C11	1.4082 (17)	C17—H17A	0.9800
C12—H12	0.9500	C17—H17B	0.9800
C3—C4	1.3602 (17)	C17—H17C	0.9800
C3—H3	0.9500	C6—H6	0.9500
C9—C8	1.4360 (17)	C18—H18A	0.9800
C11—C16	1.4125 (17)	C18—H18B	0.9800
C11—C10	1.4502 (17)	C18—H18C	0.9800
			
C3—N1—C2	130.07 (11)	C8—C10—H10	114.2
C3—N1—C7	122.91 (10)	C11—C10—H10	114.2
C2—N1—C7	106.99 (10)	N2—C7—N1	111.07 (10)
C7—N2—C1	105.09 (10)	N2—C7—C6	130.50 (11)
C14—N4—C17	119.86 (11)	N1—C7—C6	118.39 (11)
C14—N4—C18	120.26 (11)	N2—C1—C2	111.66 (11)
C17—N4—C18	117.68 (11)	N2—C1—C8	119.86 (11)
N1—C2—C1	105.19 (10)	C2—C1—C8	128.37 (11)
N1—C2—H2	127.4	C6—C5—C4	119.56 (11)
C1—C2—H2	127.4	C6—C5—H5	120.2
C13—C12—C11	122.42 (11)	C4—C5—H5	120.2
C13—C12—H12	118.8	C16—C15—C14	121.50 (12)
C11—C12—H12	118.8	C16—C15—H15	119.2
C4—C3—N1	117.18 (11)	C14—C15—H15	119.2
C4—C3—H3	121.4	C15—C16—C11	121.61 (11)
N1—C3—H3	121.4	C15—C16—H16	119.2
N3—C9—C8	179.32 (14)	C11—C16—H16	119.2
C12—C11—C16	116.65 (11)	N4—C17—H17A	109.5
C12—C11—C10	117.57 (11)	N4—C17—H17B	109.5
C16—C11—C10	125.74 (11)	H17A—C17—H17B	109.5
C10—C8—C9	122.62 (11)	N4—C17—H17C	109.5
C10—C8—C1	123.28 (11)	H17A—C17—H17C	109.5
C9—C8—C1	113.98 (10)	H17B—C17—H17C	109.5
C3—C4—C5	122.55 (11)	C5—C6—C7	119.40 (12)
C3—C4—Cl	118.87 (10)	C5—C6—H6	120.3
C5—C4—Cl	118.57 (9)	C7—C6—H6	120.3
N4—C14—C13	122.03 (11)	N4—C18—H18A	109.5
N4—C14—C15	120.91 (11)	N4—C18—H18B	109.5
C13—C14—C15	117.06 (11)	H18A—C18—H18B	109.5
C12—C13—C14	120.74 (11)	N4—C18—H18C	109.5
C12—C13—H13	119.6	H18A—C18—H18C	109.5
C14—C13—H13	119.6	H18B—C18—H18C	109.5
C8—C10—C11	131.53 (11)		
			
C3—N1—C2—C1	−177.90 (12)	C2—N1—C7—N2	0.53 (14)
C7—N1—C2—C1	0.03 (13)	C3—N1—C7—C6	0.72 (17)
C2—N1—C3—C4	177.72 (12)	C2—N1—C7—C6	−177.39 (11)
C7—N1—C3—C4	0.08 (18)	C7—N2—C1—C2	0.87 (14)
C13—C12—C11—C16	0.73 (18)	C7—N2—C1—C8	−175.75 (11)
C13—C12—C11—C10	−177.18 (11)	N1—C2—C1—N2	−0.56 (14)
N1—C3—C4—C5	−0.68 (19)	N1—C2—C1—C8	175.71 (12)
N1—C3—C4—Cl	−179.55 (9)	C10—C8—C1—N2	173.65 (11)
C17—N4—C14—C13	3.96 (19)	C9—C8—C1—N2	−2.42 (16)
C18—N4—C14—C13	166.69 (12)	C10—C8—C1—C2	−2.3 (2)
C17—N4—C14—C15	−176.41 (12)	C9—C8—C1—C2	−178.42 (12)
C18—N4—C14—C15	−13.68 (18)	C3—C4—C5—C6	0.5 (2)
C11—C12—C13—C14	0.14 (19)	Cl—C4—C5—C6	179.34 (10)
N4—C14—C13—C12	178.58 (12)	N4—C14—C15—C16	−178.52 (12)
C15—C14—C13—C12	−1.06 (18)	C13—C14—C15—C16	1.13 (18)
C9—C8—C10—C11	2.2 (2)	C14—C15—C16—C11	−0.26 (19)
C1—C8—C10—C11	−173.55 (12)	C12—C11—C16—C15	−0.67 (18)
C12—C11—C10—C8	176.01 (13)	C10—C11—C16—C15	177.05 (12)
C16—C11—C10—C8	−1.7 (2)	C4—C5—C6—C7	0.37 (19)
C1—N2—C7—N1	−0.84 (14)	N2—C7—C6—C5	−178.37 (13)
C1—N2—C7—C6	176.75 (13)	N1—C7—C6—C5	−0.92 (18)
C3—N1—C7—N2	178.64 (11)		

### (Z)-2-(6-Chloroimidazo[1,2-a]pyridin-2-yl)-3-[4-(dimethylamino)phenyl]prop-2-enenitrile Hydrogen-bond geometry (Å, º)

*Cg*1 and *Cg*3 are the centroids of the N1/C2/C1/N2/C7 and C11–C16 rings, respectively.

**Table:**

*D*—H···*A*	*D*—H	H···*A*	*D*···*A*	*D*—H···*A*
C5—H5···N3^i^	0.95	2.58	3.3871 (17)	143
C17—H17*C*···Cl^ii^	0.98	2.96	3.6211 (15)	126
C6—H6···N2^i^	0.95	2.59	3.4554 (16)	151
C16—H16···N3	0.95	2.57	3.4275 (17)	151
C3—H3···*Cg*3^iii^	0.95	2.67	3.3054 (13)	125
C12—H12···*Cg*1^iii^	0.95	2.80	3.5101 (14)	132

Symmetry codes: (i) −*x*+1, −*y*+2, −*z*+1; (ii) −*x*+1, *y*+1/2, −*z*+1/2; (iii) −*x*+1, *y*−1/2, −*z*+1/2.
